# In Ovo Injection of Betaine Affects Hepatic Cholesterol Metabolism through Epigenetic Gene Regulation in Newly Hatched Chicks

**DOI:** 10.1371/journal.pone.0122643

**Published:** 2015-04-10

**Authors:** Yun Hu, Qinwei Sun, Xiaoliang Li, Min Wang, Demin Cai, Xi Li, Ruqian Zhao

**Affiliations:** Key Laboratory of Animal Physiology & Biochemistry, Nanjing Agricultural University, Nanjing, P. R. China; Clermont Université, FRANCE

## Abstract

Betaine is reported to regulate hepatic cholesterol metabolism in mammals. Chicken eggs contain considerable amount of betaine, yet it remains unknown whether and how betaine in the egg affects hepatic cholesterol metabolism in chicks. In this study, eggs were injected with betaine at 2.5 mg/egg and the hepatic cholesterol metabolism was investigated in newly hatched chicks. Betaine did not affect body weight or liver weight, but significantly increased the serum concentration (*P* < 0.05) and the hepatic content (*P* < 0.01) of cholesterol. Accordingly, the cholesterol biosynthetic enzyme HMGCR was up-regulated (*P* < 0.05 for both mRNA and protein), while CYP7A1 which converts cholesterol to bile acids was down-regulated (*P* < 0.05 for mRNA and *P* = 0.07 for protein). Moreover, hepatic protein content of the sterol-regulatory element binding protein 1 which regulates cholesterol and lipid biosynthesis, and the mRNA abundance of ATP binding cassette sub-family A member 1 (ABCA1) which mediates cholesterol counter transport were significantly (*P* < 0.05) increased in betaine-treated chicks. Meanwhile, hepatic protein contents of DNA methyltransferases 1 and adenosylhomocysteinase-like 1 were increased (*P* < 0.05), which was associated with global genomic DNA hypermethylation (*P* < 0.05) and diminished gene repression mark histone H3 lysine 27 trimethylation (*P* < 0.05). Furthermore, CpG methylation level on gene promoters was found to be increased (*P* < 0.05) for CYP7A1 yet decreased (*P* < 0.05) for ABCA1. These results indicate that in ovo betaine injection regulates hepatic cholesterol metabolism in chicks through epigenetic mechanisms including DNA and histone methylations.

## Introduction

Betaine, which contains three chemically reactive methyl groups, is an important component of the methionine cycle which is essential for the epigenetic gene regulation through DNA and histone methylations [1,2]. Betaine homocysteine methyltransferase (BHMT) uses betaine as a substrate for converting homocysteine to methionine, and methionine adenosyltransferase (MAT) then transfers a methyl group from methionine to S-adenosylmethionine (SAM) [3], the primary methyl group donor for DNA and histone methylations catalyzed by DNA (cytosine-5-)-methyltransferases (DNMTs) and histone methlytransferases (HMTs), respectively [4]. After donating its methyl group, SAM is converted to S-adenosylhomocysteine (SAH) by glycine N-methyltransferase (GNMT), and SAH then returns to methionine cycle after being hydrolyzed back to homocysteine by S-adenosylhomocysteine hydrolase (AHCY) [5].

Betaine deficiency can cause a series of metabolic syndrome with lipid disorders [2,6], while betaine supplementation in the diet can reduce obesogenic diet-induced fatty liver in rats [7]. Betaine is used as a feed additive to improve the growth performance and carcass characteristics in livestock animals [8,9], and is also considered as an effective antioxidant agent and a methyl donor for improving the meat quality in broiler chickens [10,11]. Recently, it is reported that betaine supplementation in maternal diet can modify hepatic cholesterol metabolism in neonatal offspring piglets through epigenetic regulation of cholesterol metabolic genes [12]. Chicken eggs contain considerable amount of betaine [13], yet whether betaine in the egg affects hepatic cholesterol metabolism in newly hatched chicks remains unknown.

Cholesterol homeostasis in the liver is maintained through highly coordinated and strictly regulated biological processes including cholesterol biosynthesis, transformation and transportation [14,15]. Sterol regulatory element binding protein 1 and 2 (SREBP1 and 2) are the key transcription factors for the activation of cholesterol biosynthesis genes such as the rate-limiting enzyme 3-hydroxy-3-methylglutaryl-CoA reductase (HMGCR) [16,17]. Cholesterol can be catabolized through its transformation into excretable bile acids by cholesterol-7alpha-hydroxylase (CYP7A1) and cholesterol-27alpha-hydroxylase (CYP27A1) [18,19]. In addition, ATP-binding cassette sub-family A member 1 (ABCA1) regulates high-density lipoprotein (HDL) biogenesis and mediates the reverse cholesterol transport back to liver for disposal [20,21]. The programming effects of maternal betaine on offspring hepatic cholesterol metabolism in the pig involves epigenetic modifications of HMGCR and CYP7A1 genes [12], yet it remains to be determined whether epigenetic mechanisms are involved in the effects of betaine *in ovo*, if any, on the hepatic cholesterol metabolism in posthatch chicks.

Therefore, the objectives of the present study were to investigate the effect of *in ovo* injection of betaine on hepatic cholesterol metabolism in newly hatched chicks, and to unravel the potential mechanisms by determining the expression of genes/proteins involved in cholesterol metabolism and methyl transfer, as well as the status of DNA methylation on the promoter of affected cholesterol metabolic genes.

## Materials and Methods

### Ethics Statement

The experimental protocol was approved by the Animal Ethics Committee of Nanjing Agricultural University, with the project number 2012CB124703. The sampling procedures complied with the “Guidelines on Ethical Treatment of Experimental Animals” (2006) No. 398 set by the Ministry of Science and Technology, China.

### Animals and treatment

One hundred and forty fertilized eggs (42.1 ± 0.11 g, ranging from 39.2 g to 45.3 g) laid by Rugao yellow breeder hens were obtained from Poultry Institute of Yangzhou, Jiangsu, China, and were randomly divided into two groups (70 in each group). Before incubation, betaine (B2629, Sigma–Aldrich, USA) was dissolved in saline and injected at the dose of 2.5 mg per egg in a volume of 100 μL. The dose was determined according to the range of betaine concentrations in chicken eggs reported previously [13]. Injection was performed as previously described [22]. Eggs were injected by advancing a Hamilton syringe into a hole in the middle of the long axis until the yolk membrane was penetrated (approximately 20 mm below the surface). The incubator was set according to our previous publications [23,24]. No obvious differences in hatchability or hatching time were observed between two groups. After hatching, all the chicks were weighed and killed by rapid decapitation which is considered to be acceptable for euthanasia of small (< 200 g) birds according to American Veterinary Medical Association (AVMA) Guidelines for the Euthanasia of Animals: 2013 Edition. Blood samples were taken and serum was separated and stored at -20°C. Liver (without the gall bladder) samples were rapidly frozen in liquid nitrogen and stored at -70°C for further analysis.

### Determination of serum concentration of cholesterol and hepatic contents of cholesterol and free amino acids

Total cholesterol (Tch) in serum and in liver was measured by using commercial cholesterol assay kits purchased from Applygen Technologies Inc., China (E1005) and Beijing North of Fine Chemicals LLC, China (006301), respectively. Briefly, approximately 50 mg of liver sample was homogenized in 1 mL ice-cold buffer RIPA (18 mmol/L Tris, pH 7.5, 300 mmol/L mannitol, 50 mmol/L EDTA, 0.1 mmol/L PMSF) by a Polytron homogenizer (PT1200E, Brinkman Instruments, Littau, Switzerland). 150 μL of homogenates were mixed with 600 μL mixture of chloroform/methanol (2:1, vol/vol), then vigorously shaken for 1 min and stood for 30 min, then centrifuged at 3000 g for 10 min. The bottom (chloroform) layer was collected, air-dried and reconstituted in 30 μL mixture of tert-butyl alcohol and methanol (13:2, vol/vol), followed by the Tch determination. Hepatic concentration of free amino acids was determined in duplicate with an automatic amino acid analyzer (L-8900, Hitachi, Japan).

We measured the serum cholesterol levels for both male and female chicks, but only males showed significant change. Therefore, we focused the further analyses on male chicks. The samples of female chicks are kept in deep freezer for future research.

### Total RNA isolation and real-time PCR

Total RNA was isolated from 50 mg liver samples using 1 mL of TRIzol reagent (Invitrogen, USA). 2 μg of total RNA was treated with RNase-free DNase and reverse-transcribed to cDNA using the random hexamer primers (Promega, USA). 2 μL of diluted cDNA (1:25, vol/vol) was used for real-time PCR which was performed with a Mx3000P Real-Time PCR System (Stratagene, USA). The technical variations were normalized using β-actin as an internal control. Primers for real-time PCR (Table 1) were synthesized by Generay Biotech. Data were analyzed using the method of 2^-ΔΔCT^ [25].

**Table 1:** Nucleotide sequences of specific primers.

Target genes	GenBank accession	Primer sequences (5′ to 3′)	PCR products (bp)
SREBP1	AY029224	F: CTACCGCTCATCCATCAACG	145
		R: CTGCTTCAGCTTCTGGTTGC	
SREBP2	XM_416222	F: CCCAGAACAGCAAGCAAGG	108
		R: GCGAGGACAGGAAAGAGAGTG	
HMGCR	NM_204485.1	F: TTGGATAGAGGGAAGAGGGAAG	137
		R: CCATAGCAGAACCCACCAGA	
CYP7A1	AB109636.1	F: CATTCTGTTGCCAGGTGATGTT	106
		R: GCTCTCTCTGTTTCCCGCTTT	
CYP27A1	XM422056.4	F: AGGACTTTCGTCTGGCTCT	185
		R: CTCCGCATCGGGTATTT	
ABCA1	NM_204145.2	F: TCCTCTGGCTTAGACTTGA	130
		R: CTCGTAGTTGTATTCGGTAA	
APO-A1	NM_205525.4	F: GTGACCCTCGCTGTGCTCTT	217
		R: CACTCAGCGTGTCCAGGTTGT	
LCAT	NM_001293094.1	F: CTGGTGAACAACGGCTACG	159
		R: GTGCCCAATGAGGAAGACA	
LDLR	NM_204452.1	F: CCACCATTTGGCAGAGGAA	86
		R: ACCGCAGTCAGACCAGAAGAG	
β-actin	L08165.1	F: TGCGTGACATCAAGGAGAAG	300
		R: TGCCAGGGTACATTGTGGTA	
CYP7A1 promoter	NC_006089.3	F: GGCTTCGTCCAGAAAT	150
		R: CAAACCTGCTAACTAACACTA	
ABCA1 promoter	NC_006127.3	F: CCAGGAAAGTGGTGGAGTC	174
		R: TCACAGGATAGAGGCACAGAA	
HMGCR promoter	NC_006127.3	F: GGACTCAGGGTCTAAAG	75
		R: ACAAACATTGCTCACAG	
SREBP-1 promoter	NC_006101.3	F: GGGGAGGAGGCGAGAAAA	101
		R: ACGAGGAGGAGCAGGGGTA	
SREBP-2 promoter	NC_006088.3	F: CAAGGAGATCCGCAAGGG	137
		R: GCCGCATCGGCTGAAAA	

*SREBP 1 and 2*, sterol regulatory element binding protein 1 and 2; *HMGCR*, 3-hydroxy-3-methyl-glutaryl coenzyme A reductase; *CYP7A1*, cholesterol-7-alpha hydroxylase; *CYP27A1*, sterol 27-hydroxylase; *APO-A1*, apolipoprotein A-1; *LCAT*, lecithin-cholesterol acyltransferase; *LDLR*, low-density lipoprotein receptor.

### Total protein extraction and western blotting

Total protein were extracted from 100 mg frozen liver sample as previously described [26]. Protein concentrations were determined using Pierce BCA Protein Assay kit (Rockford, IL, USA) according to the manufacturer’s instructions. 40 μg of protein was used for electrophoresis on a 10% or 7.5% SDS-PAGE gel. Western blot analysis for HMGCR (bs6625, Bioworld Technology, USA, diluted 1:1000), SREBP1 (sc-366, santa cruz, USA, diluted 1:200), SREBP2 (sc-5603, santa cruz, USA, diluted 1:200), CYP7A1 (ab79847, Abcam, UK, diluted 1:200), CYP27A1 (bs2192, Bioworld Technology, USA, diluted 1:200), BHMT (15965-1-AP, Proteintech, USA, diluted 1:200), AHCYL1 (10658-3-AP, Proteintech, USA, diluted 1:1000), DNMT1 (24206-1-AP, Proteintech, USA, diluted 1:1000), DNMT3a (bs6587, Bioworld Technology USA, diluted 1:500), H3K27me3 (17–622, Millipore, USA, diluted 1:500) was carried out according to the recommended protocols provided by the manufacturers. The β-actin (AP0060, Bioworld, USA, diluted 1:10,000) was used as loading control in the Western blot analysis. Images were captured by VersaDoc 4000MP system (Bio-Rad, USA) and the band density was analyzed with Quantity One software (Bio-Rad, USA).

### Slot blotting and South-western analysis

High-quality genomic DNA was isolated from liver tissues. DNA concentrations was measured with NanoDrop ND-1000 Spectrophotometer (Thermo, USA). 4 μg of DNA was transferred onto the nitrocellulose membranes (BioTrace, Pall Co, USA), cross-linked by baking at 80°C for 1 h, blocked at room temperature for 2 h and then incubated with 5-methyl cytidine antibody (ab10805, Abcam, UK, 1:500) overnight at 4°C. After three washes in Tris-Buffered-Saline with Tween (TBST), membranes were incubated with goat anti-mouse horseradish peroxidase (HRP)-conjugated secondary antibody (1:10,000; Bioworld, USA) at room temperature for 2 h. The methods of image capture and data analysis followed that of western blotting.

### Methylated DNA immunoprecipitation (MeDIP) analysis

High-quality genomic DNA was isolated from liver tissues and sonicated to fragments with the size of approximately 500 bp. 2 μg of fragmented DNA was heat-denatured to produce single-stranded DNA, and a portion of the denatured DNA was stored as input DNA. A mouse monoclonal antibody against 5-methyl cytosine (ab10805, Abcam, UK) was used to immunoprecipitate methylated DNA fragments. The immune complexes were captured with protein G agarose beads pretreated with denatured salmon sperm DNA and BSA (No. P2009, Beyotime Institute of Biotechnology, china). The beads bound to immune complexes were washed to eliminate nonspecific binding and resuspended in 250 μL digestion buffer containing proteinase K. Finally, the MeDIP DNA was purified. A small aliquot of MeDIP DNA and control input DNA was used to amplify the proximal promoter sequence of chicken CYP7A1, ABCA1, HMGCR, SREBP1 and SREBP2 genes by real-time PCR with specific primers listed in Table 1. Data was normalized against the input and presented as the fold change relative to the average value of the control group.

### Statistical analysis

The results are presented as means ± SEM. Comparisons were performed using independent-samples T-Test with SPSS 20.0 for windows. The differences were considered statistically significant when *P* < 0.05.

## Results

### Body weight, liver weight, serum cholesterol concentration, hepatic content of cholesterol and free amino acids

As shown in Table 2, *in ovo* injection of betaine did not significantly affect the body weight or the liver weight of newly hatched chicks. However, chicks hatched from betaine-injected eggs had significantly higher cholesterol concentration in liver (*P* < 0.01) and in serum (*P* < 0.05). Based on the consideration that betaine is involved in the hepatic amino acids metabolism, and betaine intake increased the hepatic serine levels in mice [27], we detected the hepatic content of free amino acids. Betaine-treated chicks had significantly higher (*P* < 0.05) content of free serine in the liver, as compared with their control counterparts (Table 3).

**Table 2:** Body weight and liver weight, serum concentration and hepatic content of total cholesterol in newly hatched chicks.

Parameters	Control (n = 8)	Betaine (n = 8)	*P*-value
BW (g)	30.8 ± 0.6	29.4 ± 0.5	0.08
LW (mg)	702.5 ± 18.6	696.9 ± 20.5	0.70
Serum Tch (mmol/L)	9.3 ± 0.9	12.1 ± 0.7	0.02
Hepatic Tch (mg/g)	10.1 ± 1.0	13.5 ± 0.6	0.005

Values are means ± SEM, n = 8. BW, body weight; LW, liver weight; Tch, total cholesterol.

**Table 3:** The hepatic content of free amino acids.

Amino acids (mg/100 g)	Control (n = 4)	Betaine (n = 6)	*P*-value
Asp	21.3 ± 0.9	20.5 ± 0.8	0.54
Thr	41.5 ± 0.5	42.7 ± 1.7	0.61
Ser	29.3 ± 1.3	33.3 ± 0.8	0.02
Glu	54.0 ± 2.6	51.3 ± 1.0	0.30
Gly	19.3 ± 1.8	19.3 ± 0.6	0.96
Ala	15.5 ± 1.6	15.7 ± 0.5	0.91
Cys	7.3 ± 0.6	6.3 ± 0.2	0.14
Val	15.3 ± 1.0	15.2 ± 0.5	0.93
Met	7.0 ± 0.6	6.8 ± 0.2	0.75
Ile	7.0 ± 0.7	7.1 ± 0.3	0.81
Leu	15.8 ± 1.6	16.5 ± 0.6	0.62
Tyr	10.5 ± 0.9	10.7 ± 0.2	0.83
Phe	9.0 ± 0.9	9.7 ± 0.3	0.70
Lys	22.6 ± 2.5	20.8 ± 2.0	0.56
His	9.3 ± 0.5	9.5 ± 0.3	0.67
Arg	17.3 ± 2.4	17.7 ± 1.2	0.87
Pro	6.7 ± 1.8	7.0 ± 0.4	0.87

Values are means ± SEM, n = 4 or 6. Asp = Aspartate; Thr = Threonine; Ser = Serine; Glu = Glutamate; Gly = Glycine; Ala = Alanine; Cys = Cysteine; Val = Valine; Met = Methionine; Ile = Isoleucine; Leu = Leucine; Tyr = Tyrosine; Phe = Phenylalanine; Lys = Lysine; His = Histidine; Arg = Arginine; Pro = Proline.

### Hepatic mRNA abundance of genes involved in cholesterol metabolism

**Fig 1:**
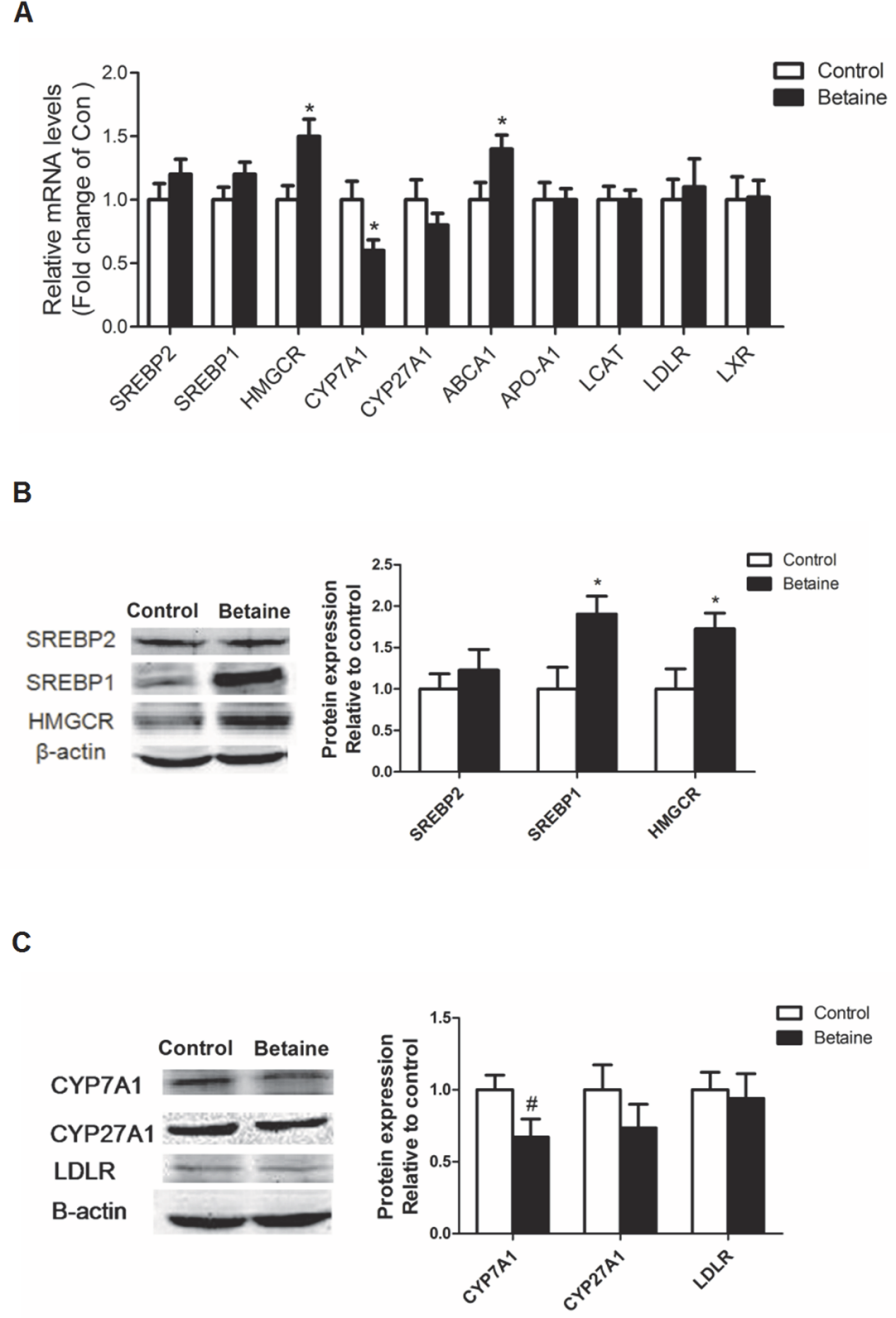
Effect of in ovo injection of betaine on hepatic expression of cholesterol metabolic genes in newly hatched chicks. A) Hepatic mRNA abundance of genes involved in cholesterol metabolism; B) Protein expression of SREBP1, SREBP2 and HMGCR; C) Protein expression of CYP7A1, CYP27A1 and LDLR. Values are means ± SEM, *P < 0.05, compared with control (n = 8).

As shown in Fig 1A, the rate-limiting cholesterol biosynthesis enzyme HMGCR, as well as ABCA1 which mediates the cholesterol counter transport, were significantly up-regulated (*P* < 0.05) at the level of mRNA in the liver of betaine-treated chicks, which coincided with significantly down-regulated (*P* < 0.05) cholesterol catabolic gene CYP7A1. No significant alterations (*P* > 0.05) were detected for other cholesterol metabolic genes, including SREBP1, SREBP2, CYP27A1, APO-A1, LDLR, LCAT and LXR at the level of mRNA, in the liver of newly hatched chicks.

### Hepatic protein content of enzymes involved in cholesterol metabolism

As shown in Fig 1B, the hepatic content of HMGCR protein was significantly increased (*P* < 0.05), which was in line with its up-regulated mRNA level in betaine-treated group. Although the mRNA level of SREBP1 was not affected by betaine treatment, its protein content was significantly higher (*P* < 0.05). The protein content of CYP7A1 tended to decrease (*P* = 0.07) in the liver of betaine-treated chicks (Fig 1C). No significant changes were detected for the hepatic protein content of SREBP2, CYP27A1, and LDLR (Fig 1B and 1C). We failed to detect ABCA1 protein in Western blot analysis, because there was no specific antibody available to detect chicken ABCA1.

### Hepatic protein content of enzymes involved in methyl transfer and methylation status of global genomic DNA and histone H3

**Fig 2:**
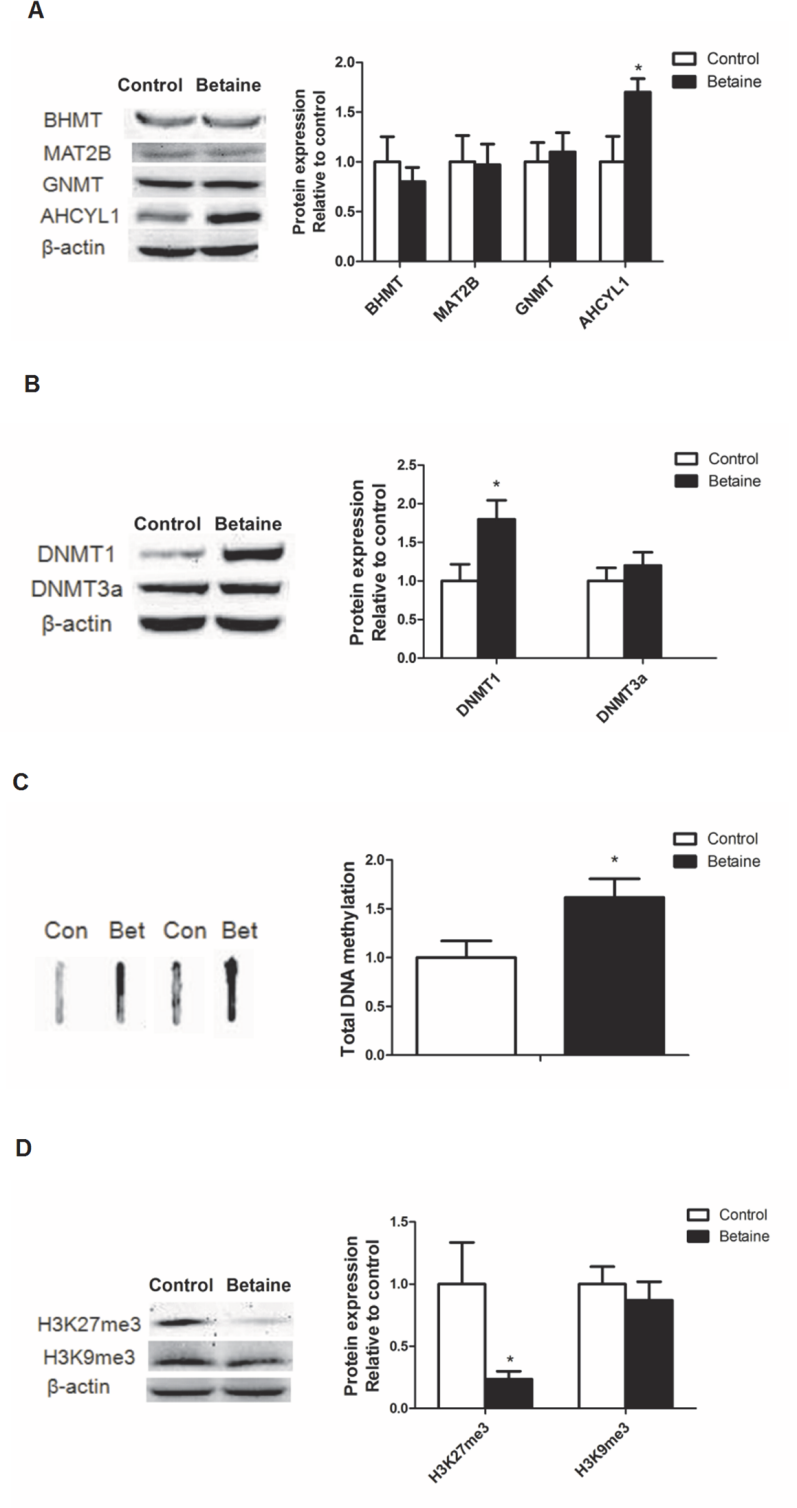
Effect of in ovo injection of betaine on hepatic protein content of enzymes involved in methyl transfer and methylation status of global genomic DNA and histone H3. A) Protein expression of BHMT, MAT2B, GNMT and AHCYL1; B) Protein expression of DNMT1 and DNMT3a. C) Total DNA methylation in liver; D) Protein expression of H3K27me3 and H3K9me3. Values are means ± SEM, *P < 0.05, compared with control (n = 8).

*In ovo* injection of betaine significantly up-regulated the protein level of AHCYL1 (*P* < 0.05), which hydrolyzes SAH back to homocysteine in the methionine cycle (Fig 2A). Moreover, the protein content of DNMT1 (*P* < 0.05) was significantly increased in the liver of betaine-treated chicks (Fig 2B), which was accompanied by increased (*P* < 0.05) global genomic DNA methylation (Fig 2C), yet diminished (*P* < 0.05) gene repression mark histone H3 lysine 27 trimethylation (H3K27me3) (Fig 2D).

### MeDIP analysis for DNA methylation status on gene promoters

**Fig 3:**
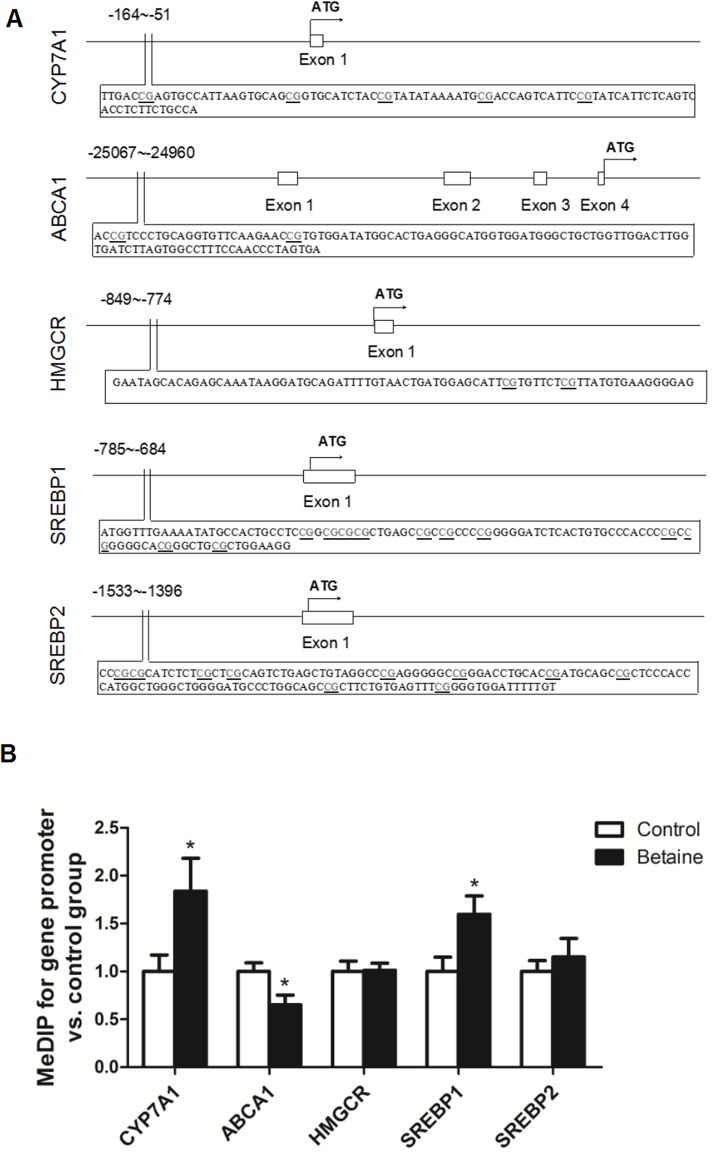
Methylation status on the promoter of CYP7A1, ABCA1, HMGCR, SREBP1 and SREBP2 genes in the liver of newly hatched chicks. A) Schematic diagram showing the promoter sequences of chicken CYP7A1, ABCA1, HMGCR, SREBP1 and SREBP2 genes CpG sites, determined in this study, on 5’-flanking promoter regions of CYP7A1 (-164~-51), ABCA1 (-25067~-24960), HMGCR (-849~-774), SREBP1 (-785~-684) and SREBP2 (-1533~1396) relative to the translation start codon (ATG) are underlined. B) Methylation status on the promoter of CYP7A1, ABCA1, HMGCR, SREBP1 and SREBP2 genes. Values are means ± SEM, *P < 0.05, compared with control (n = 6).

The promoter sequences of chicken CYP7A1, ABCA1, HMGCR, SREBP1 and SREBP2 genes determined for the methylation status in MeDIP analysis are shown schematically in Fig 3A. As shown in Fig 3B, CYP7A1 promoter was significantly hypermethylated (*P* < 0.05), while ABCA1 promoter was significantly hypomethylated (*P* < 0.05) in the liver of betaine-treated chicks, which were reversely correlated, respectively, with the mRNA abundances of these two genes. SREBP1 promoter was significantly hypermethylated, while no alterations were detected in the methylation status of HMGCR and SREBP2 promoters in the MeDIP analysis.

## Discussion

Previous studies concerning the effects of betaine on cholesterol metabolism have been predominately carried out on adult human subjects [28,29] or animals [30–32]. Betaine is commonly perceived as an important nutrient for embryonic and fetal development [2], whereas the effects of prenatal betaine supplementation on cholesterol homeostasis in neonatal animals are less understood. Previously, we reported that dietary betaine supplementation in gestational sows increased hepatic cholesterol accumulation in neonatal piglets, while the serum cholesterol profiles were not affected [12]. In this study, *in ovo* administration of betaine increased both serum concentration and hepatic content of cholesterol in newly hatched chicks, thus providing new evidence of betaine’s fetal programming effects on hepatic cholesterol metabolism in the chicken.

To date, majority of the betaine efficacy studies have been restricted to phenotypic descriptions. In the present study, the cholesterol-elevating effects of prenatal betaine exposure in the chicken were associated with modulated expression of cholesterol metabolic genes. Similar to what we found in neonatal piglets [12], hepatic expression of HMGCR and CYP7A1 were modulated by prenatal betaine administration also in newly hatched chicks in this study. The up-regulation of HMGCR, together with higher hepatic content of SREBP1 protein, indicate enhanced cholesterol biosynthesis in the liver, while the down-regulation of CYP7A1 implicates suppressed cholesterol conversion or transformation to bile acids. The combined effects of enhanced cholesterol biosynthesis and suppressed cholesterol catabolism may contribute to higher cholesterol accumulation in the liver of betaine-treated chicks. Moreover, ABCA1 which regulates HDL biogenesis and mediates the cholesterol counter transport [21] was up-regulated, at the level of mRNA, in the liver of betaine-treated chicks. Although the hepatic protein content of ABCA1 was not determined due to lack of specific antibody, the transcriptional activation of ABCA1 gene is presumably in accordance with increased hepatic cholesterol content.

SREBP2 is the main regulator of cholesterol synthesis and metabolism in both mammalian and avain species, therefore it is surprising that SREBP1, but not SREBP2, was modulated in betaine-treated chicken liver. In mammals, SREBP1 gene produces two different isoforms, SREBP-1a and -1c, which differ in their first exons by using different transcriptional start sites. SREBP1a regulates both cholesterol and fatty acid biosynthesis, whereas SREBP1c mainly regulates fatty acid and glucose metabolism. In chickens, however, only one transcript of chicken SREBP1 gene has been identified which is highly homologous to the SREBP1a in mammals [33].

The biological activities of betaine are presumed to be attributable to its methyl groups [34], however the direct evidences to support this notion are scarce. In the present study, *in ovo* injection of betaine significantly increased hepatic content of serine, a major metabolite of methionine cycle and one carbon metabolism [35]. Moreover, *in ovo* injection of betaine significantly up-regulated protein content of AHCYL1 and DNMT1 in the liver of newly hatched chicks, which agrees with a previous finding that maternal betaine supplementation causes DNMT1 up-regulation in the hippocampus of neonatal piglets [34]. DNMT1 is the most abundant DNA methyltransferase in mammalian cells responsible for maintaining the DNA methylation pattern during cell division [36]. DNMT1 overexpression generally leads to increased global DNA methylation [37]. Indeed, the up-regulation of DNMT1 in the present study was associated with global DNA hypermethlylation in the liver of betaine-treated chicks.

The effects of methyl donors on DNA methylation have been well documented [38], yet how these dietary factors regulate histone methylations is less understood. The expression of histone methyltransferases G9a and Suv39h1 was reported to be directly related to the dietary content of choline, and the level of H3K27me3, a transcriptional repression mark, was up-regulated by choline supplementation in rats [39]. In the present study, we were not able to detect the expression of histone methyltransferases due to lack of antibodies, but the level of H3K27me3 was decreased in the liver of betaine-treated chicks. A number of factors may contribute to the disparity, which include differences in animal species, type of the methyl nutrients, the timing, dose, duration or the route of supplementation, just to name a few.

It is noted that DNA methylation machinery responds to methyl donor availability in a complex fashion, the methylation status of the promoters or regulatory regions of specific genes may respond differently from that of the global genomic DNA [40]. In fact, the alterations of promoter methylation in response to *in ovo* betaine administration are gene-specific in the present study. The promoter of CYP7A1 gene was found to be hypermethylated, while that of ABCA1 gene was hypomethylated, being reversely linked to the transcriptional repression of CYP7A1 gene and transcriptional activation of ABCA1 gene, respectively, in the liver of betaine-treated chicks. Nevertheless, transcriptional regulation of gene expression is complex and mismatches between the methylation level of the promoter and the mRNA abundance of the gene were also observed. For instance, SREBP-1 promoter was significantly hypermethylated, whereas no alteration was detected for its mRNA abundance. HMGCR mRNA was significantly up-regulated yet no alteration was detected in the methylation status of its promoter which contains very few CpG sites.

In conclusion, we demonstrate, for the first time, that *in ovo* injection of betaine induces cholesterol accumulation in the liver of newly hatched chicks through epigenetic regulation of cholesterol metabolic genes in the liver. Presently, it is not possible to relate such neonatal alterations in hepatic cholesterol metabolism to health benifits or disease risks in later life. Follow-up studies are required to elucidate whether altered methylation patterns and cholesterol homeostasis in newly-hatched chicks may lead to long-term deregulation of cholesterol metabolism in adult chickens, and to evaluate whether the performance of broiler chickens may be improved through prenatal programming of cholesterol homeostasis by dietary manipulation of betaine in breeder hens.
