# Correction: In Ovo Injection of Betaine Affects Hepatic Cholesterol Metabolism through Epigenetic Gene Regulation in Newly Hatched Chicks

**DOI:** 10.1371/journal.pone.0130786

**Published:** 2015-06-11

**Authors:** The PLOS ONE Staff

The second author and the fifth author, Qinwei Sun and Ruqian Zhao, should both be noted as corresponding authors of this work. Ruqian Zhao can be contacted at zhao.ruqian@gmail.com. The publisher apologizes for this error.
